# Immunohaematological Testing, Monitoring, and Treatment of Haemolytic Disease of the Newborn: Where are we in this Field after 25 Years?

**DOI:** 10.34763/jmotherandchild.20263001.d-26-00007

**Published:** 2026-09-25

**Authors:** Marjana Jerković Raguž, Sanja Klarić, Mirela Mabić

**Affiliations:** Department of Neonatology, Clinic for Children’s Diseases, University Clinical Hospital Mostar,Bosnia and Herzegovina; School of Medicine, University of Mostar, 88000 Mostar, Bosnia and Herzegovina; Institute of Transfusion Medicine, University Clinical Hospital Mostar,Bosnia and Herzegovina; University of Mostar Faculty of Economics, 88000 Mostar, Bosnia and Herzegovina

**Keywords:** infants, pregnancy, haemolytic disease, sensitisation

## Abstract

**Context:**

Prevention of pregnancy-related alloimmunisation and the management of haemolytic disease of the foetus and newborn (HDFN) has significantly improved over the past decades.

**Aim:**

Considering the improved HDFN care in recent years, the goal of this 25-year analysis is to determine the epidemiological aspects of alloimmunisation during pregnancy and the presentation, diagnosis, and treatment of different types of haemolytic disease of newborns (HDN).

**Materials and methods:**

The retrospective study encompasses all pregnant women whose immunohaematological screening detected antibodies against any of the antigens in ABO, Rhesus, Kell, Duffy, Kidd, and/or other blood group systems or had newborns who developed HDN.

**Results:**

Over the 25-year study period, 830/38,898 (2.1%) of immunohaematologically tested pregnant women developed alloimmunisation, while 556 (1.42%) of their newborns developed HDN. The most common form of HDN in our study was ABO HDN at 73.2% (407/556). RhD HDN was treated in 12.9% (72/556) of the newborns. Exchange transfusions (ET) were administered to 5.76% (32/556) of newborns with HDN. ET was performed on 24 (75%) with ABO HDN, and 8 (25%) with RhD HDN.

The conclusion of this 25-year study is that alloimmunisation is still present, as are its consequences for the newborn. Despite better screening of pregnant women, diagnosis, and treatment of the consequences of alloimmunisation, we must know that there is no universal cure for HDN; therefore, continuous research and new guidelines in this field are needed.

## Introduction

Haemolytic disease of the foetus and newborn (HDFN) is an immune-mediated disorder that affects infants worldwide [1,2]. The pathophysiology of HDFN begins with maternal antibodies attacking foetal red blood cells following alloimmunisation caused by antigens from the Rh, ABO, or other systems that have been discovered in recent years [3,4]. Depending on the antigenicity and amount of antibodies involved, transplacental transmission can lead to HBFN with complications such as anaemia, jaundice, and in more severe cases, hydrops fetalis or hyperbilirubinemia, and kernicterus in the newborn [5]. Immunohaematological monitoring is an important tool for predicting potential risk. Over the last decade, thanks to immunohaematological testing, the outcomes of alloimmunisation have significantly improved in terms of preventing the most severe clinical pictures of HDFN and its consequences for both mother and child [6]. The assumption is that the spectrum of haemolytic disease of the newborn (HDN) types will change significantly in the future [7], as will the approach to sensitised pregnant women, due to increasing awareness among healthcare professionals of screening RhD-negative pregnant women and the use of anti-D prophylaxis [1]. Although there is no universal cure for HDN, neonatal treatment of HDN due to sensitisation has also developed in recent years. Therefore, interventions aim to prevent and minimise the adverse effects of associated complications. Due to its high incidence and nature, HDN has been extensively studied, with new research being conducted every year that reveals new insights into the condition [4]. ABO incompatibility is currently considered the most common single cause of HDN in the Western world [8]. Despite significant advancements in the understanding and management of HDFN, the condition remains a significant cause of perinatal morbidity and mortality if left undiagnosed and untreated. Therefore, enhanced knowledge of the pathogenesis, prevalence, prevention, and management strategies of HDFN, as well as the importance of timely intervention and the role of evolving diagnostic techniques in improving outcomes for affected pregnancies [9]. The goal of this 25-year analysis is to investigate the epidemiological aspects of maternal sensitisation to foetal red blood cell antigens (ABO, Rhesus, Kell, Duffy, and others) and to identify the most common types of HDN in the Western Herzegovina and Herzegovina-Neretva cantons of Bosnia and Herzegovina, both of which are under the jurisdiction of the Maternity Hospital at the University Clinical Hospital (UCH) Mostar.

## Subjects and Methods

The research was conducted as a retrospective epidemiological analysis at the Department of Transfusion Medicine and the Neonatal Intensive Care Unit (NICU) of the UCH Mostar, from 2000 to the end of 2024. All pregnant women who were permanent residents of the West-Herzegovina and Herzegovina-Neretva cantons in Bosnia and Herzegovina, both of which are within the jurisdiction of the Maternity Hospital of the UCH Mostar, underwent prenatal immuno-haematological screening.

The study encompasses all the pregnant women whose immunohaematological screening detected antibodies against any of the antigens in ABO, Rhesus, Kell, Duffy, Kidd, and/or other blood group systems. The study included pregnant women with the following features: 1) a positive indirect antiglobulin test (IAT) during pregnancy; 2) positive IAT at delivery; and 3) whose newborn infants had a positive direct antiglobulin test (DAT). The IAT detects antibodies that float freely in the blood of pregnant women. The DAT in newborns determines whether red blood cells (RBCs) circulating in the bloodstream are covered with antibodies.

The Department of Transfusion Medicine UCH Mostar gathered the relevant data for the study as follows: immunohaematological screening of all pregnant women (RhD positive and RhD negative) was conducted at 12 to 16 weeks of pregnancy, at 24 to 26 weeks, and after 35 weeks. If the anti-erythrocyte antibody test was positive, the screening was subsequently performed once a month, and then once every two weeks after week 28. The following parameters were analysed in the mothers included in the study: parity, ABO blood group; RhD factor; and the type, specifics, and antibody titer. The study included all the newborn infants who developed HDN caused by any of the antigens in the period under analysis. The pregnant women who did not experience maternal sensitisation were excluded from the study. The study also excluded pregnant women whose medical history showed that they had been given anti-D prophylaxis.

The study also included newborns whose antibodies were detected at birth; newborns with positive DAT and HDN whose mothers had not tested positive on the IAT during screening; and newborns whose mothers had not undergone regular screenings and whose antibodies were accordingly unidentified. Newborn infants who were treated for cholestatic jaundice or physiological jaundice were not included in the study. The data were collated from medical histories, documentation, protocols, and letters of discharge, all of which are available in the medical archives of the NICU of the UCH Mostar. For newborn infants, the parameters were: gestational age, gender, DAT, bilirubin, type of treatment, and the clinical features of HDN (mild, moderate, severe) [10].

All newborn infants with HDN were treated with supportive therapy (plasma, immunoglobulin) and phototherapy. Where necessary, ET was performed. ET is a procedure for the exchange of blood through the umbilical vein via a plastic catheter. ET or blood exchange can correct anaemia, lower bilirubin concentrations, and remove antibodies against erythrocytes from the baby’s blood. We partially follow the guidelines of the American Academy of Pediatrics (AAP), who recommend ET if bilirubin levels exceed 428 µmol/L in healthy term infants and 342 µmol/L in healthy, preterm newborns (<38 weeks) [10].

The pregnant women gave venous blood samples in order to determine blood group; RhD antigen; and type, specifics, and antibody titer at the Department of Transfusion. The optimal blood sample for erythrocyte blood-group testing for the presence of antibodies in serum and to determine the specificity of antibodies is 5–10 ml of blood in a sterile tube with no anticoagulant. Any newborn infant whose mother had a positive IAT had umbilical cord blood samples taken at birth, which were tested for ABO blood group, Rh factor, and DAT. Newborns with HDN treated at the NICU had further blood samples taken to determine blood count and bilirubin. The blood count and bilirubin tests were conducted by the Department of Laboratory Diagnostics at UCH Mostar.

## Statistical Analysis

Results are presented as frequencies and percentages, and laboratory parameters are expressed as means and standard deviations. Differences in the distribution of pregnant women and newborns according to different characteristics were tested using the Chi-square test. The significance level was set at 0.05, and p-values that could not be expressed to three decimal places are shown as p < 0.001. Data were analysed with IBM SPSS for Windows, version 25.0 (IBM Corp., Armonk, NY, USA).

## Results

From 2000 to 2024, a total of 38,898 pregnant women underwent testing at the Department of Transfusion Medicine at UCH Mostar. The research comprised 830 pregnant women (2.1%) who exhibited sensitisation. Throughout the research period, a total of 39,167 newborns were delivered at the Clinic for Gynecology and Obstetrics of the UCH Mostar. During the 25-year study period, 556 of the newborns (1.42%) were diagnosed with HDN and received treatment at the Neonatology Department of the Clinic for Pediatric Diseases. Among the pregnant women, 269 (32.4%) were primiparas, and 321 (38.7%) were second-time mothers, for a total of 590 (71.1%) women who were positive in the first two pregnancies, while the remaining 240 respondents (28.9%) had parity of 3 or more.

**Graph 1.**
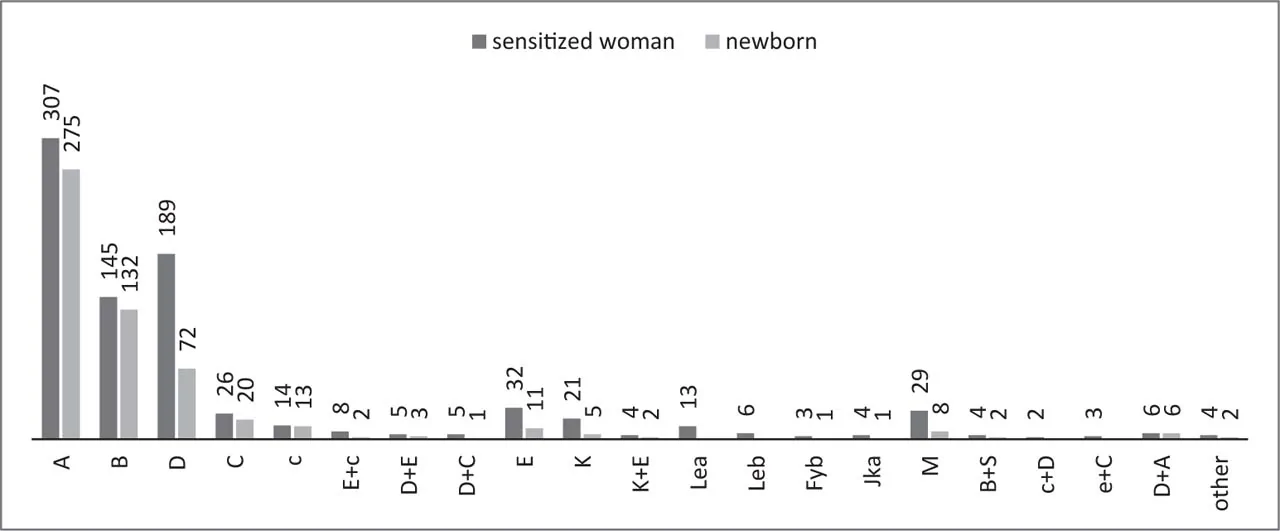
Illustrates the number of pregnant women and newborns with HDN based on the type of sensitisation from 2000 to the end of 2024.

**Graph 2.**
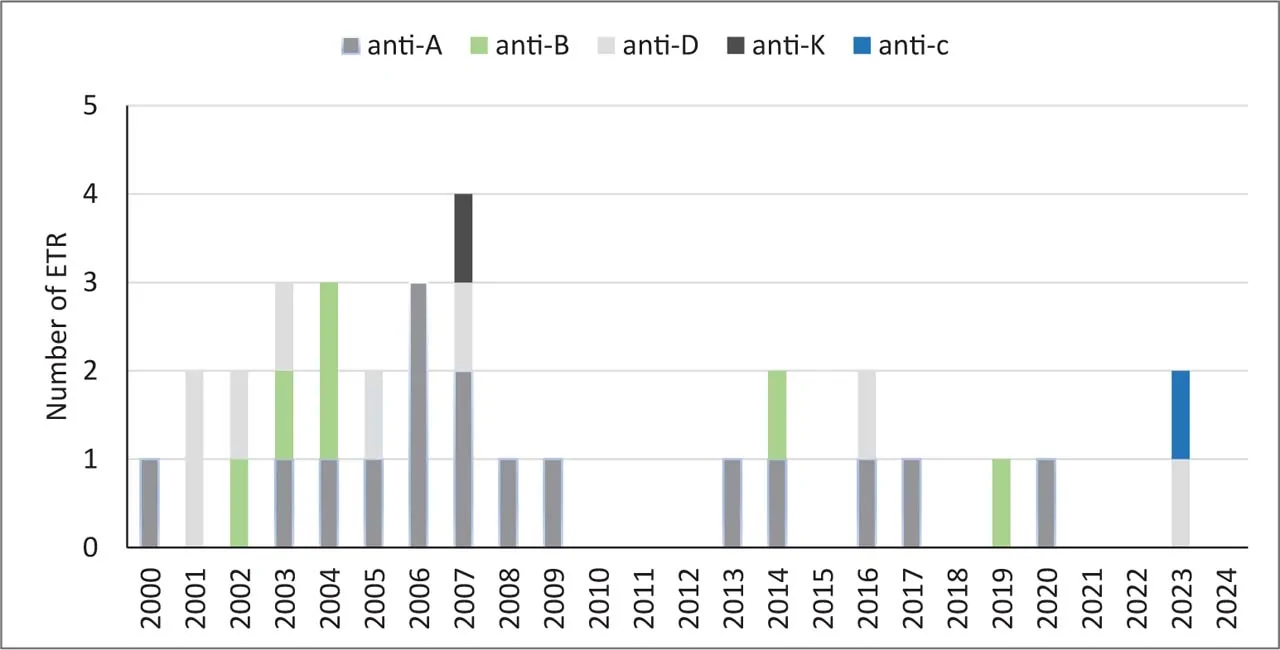
Distribution of ET on HDN causes during the period from 2000 to the end of 2024.

**Table 1.** Gestational age and mean concentration of individual laboratory parameters in newborns with HDN who underwent ET during the period from 2000 to the end of 2024.

**Antibodies**	**number**	**Gestational age (week)**		**Mean ± Standard deviation**			**%**
		
		**<37**	**37^+6/7^-42**	**Concentration of bilirubin (NV: 0–200 µmol/L)**	**Number of erythrocytes (NV: 3.9-5.5 × 10^12^/L)**	**Concentration of haemoglobin (NV: 136–199 g/L)**	**Reticulocyte**
Anti-A	16	1	15	386.4 ± 23.1	3.9 ± 0.5	127.9 ± 21.7	75
Anti-B	6		6	352.1 ± 5.2	4.6 ± 0.4	144.2 ± 4.8	35
Anti-D	8		8	237.2 ± 13.9	3.9 ± 0.3	139.4 ± 3.4	47
Anti-K	1		1	342	3.7	108	9
Anti-c	1	1		300	1.8	73	8

NV - normal values.

According to the time of detection, the final phase of pregnancy or childbirth itself predominates (590, 71.1%), of which detection occurred at childbirth in 371 (44.7%) pregnant women and after the 35th week in 219 (26.4%) pregnant women. In contrast, early detection (12-14 weeks) was present in 113 pregnant women (13.6%).

Regarding the antibody titer, the largest number of pregnant women had an undetermined status (unsounded) with 284 (34.2%), while 259 (31.2%) had extremely low titers. Among the other measured values, the titer 1:18, recorded in 180 (21.7%) pregnant women, is clearly dominant, while all other individual titers are represented at very low levels, each less than 5%.

ABO antibodies were detected at birth in 71.9% (325/452) of cases. Among the 556 newborns diagnosed with HDN, 225 (71%) presented with a moderate clinical manifestation of the condition. All neonates received phototherapy treatment. Immunoglobulin was provided to individuals with moderate and severe haemolytic disease of the newborn. During the study period, 4.6% (26/556) of pregnant women with newborns who tested positive for a direct antiglobulin test (DAT) and developed haemolytic disease of the newborn (HDN) had not received immunohaematological screening.

Among the infants with HND, 283 (50.9%) were girls and 273 (49.1%) were boys (*p* = 0.671). There were significantly more term infants, 494 (88.8%) versus 62 (11.2%) infants born before 37 weeks (*p* < 0.001).

Among the examined pregnant women, 22.7% (189/830) exhibited RhD sensitisation, while 54.4% (452/830) had ABO sensitisation. Throughout the examined timeframe, the incidence of pregnant women with RhD sensitisation markedly diminished. Our findings indicate that RhD was the predominant form of sensitisation from 2000 to 2007, with 131 cases documented; however, in the subsequent 17 years, only 58 cases of RhD sensitisation during pregnancy were identified, reflecting a significant decline. Non-RhD antibodies were produced in 77.2% (641 out of 830) of the sensitised women. Throughout the research period, a total of 39,167 newborns were delivered at the Clinic for Gynecology and Obstetrics of the UCH Mostar. During the 25-year study period, 556 newborns (1.42%) diagnosed with HDN received treatment at the Neonatology Department of the Clinic for Pediatric Diseases. In our study, the predominant form of HDN was ABO HDN, accounting for 73.2% (407/556) of cases. RhD HDN was treated in 12.9% of the newborns (72 out of 556). Seventy-seven newborns (13.8%) experienced non-RhD HDN attributed to antibodies (c, C, E, M, K).

Exchange transfusions were administered to 5.76% (32/556) of newborns with HDN, comprising 16 cases of anti-A, 6 cases of anti-B, 8 cases of anti-D, 1 case of anti-K, and 1 case of anti-c. The primary cause of ET was ABO incompatibility, specifically anti-A antibodies, present in 50% (16/32) of the newborns undergoing the procedure. The findings indicate a decline in the number of ET over the years, with 32% (10/32) of the ET conducted in the latter half of the study period. The findings indicate a significant decline in the number of ET over the years, with 31.2% (10/32) of the ET conducted in the latter half of the study period compared to 22 (68.8%) in the first half of the observed period (*p* = 0.034).

Newborns with HDN caused by RhD antibodies underwent ET in the first 24 hours of life because they had a high concentration of bilirubin. Newborns with HDN ABO had a high concentration of bilirubin from 48 to 96 hours of life that required ET.

## Discussion

Sensitisation during pregnancy and its consequences for newborns still account for a significant portion of neonatal pathology [9]. Although a better understanding of the immunisation process and improved communication between gynaecologists and transfusion specialists have contributed to raising awareness about the importance of performing screening three times during pregnancy and the early recognition of RhD and non-RhD immunisation in pregnant women [1], as proven by the results of this study in recent years. However, some countries still struggle with RhD immunisation, with a 2023 study reporting that approximately 70% of severe RhD HDN cases occurred despite anti-D HDN being largely preventable [11]. Unfortunately, some countries do not even perform immunological screening on all pregnant women, due to a lack of comprehensive healthcare, unfamiliarity with the process of alloimmunisation in pregnancy, or an inability to access a reference transfusion centre [12]. We are far from eradicating HDN on a global scale and from universally treating those who have already developed HDN.

However, due to its high incidence and nature, HDN has been extensively studied, with ongoing research revealing new insights into the condition, such as that ABO incompatibility is currently the most common single cause of HDN in the Western world [7]. Thanks to the long-term monitoring of HDN in our region, we can conclude that the spectrum of HDN has changed, and with it, the severity of the clinical picture in newborns. Our results show that 42% (230/545) of pregnant women developed ABO sensitisation, confirming that ABO incompatibility is now synonymous with HDN [13]. Immunohaematological screening of pregnant women was nearly 100% in our study, contributing to earlier diagnosis and monitoring of newborns from birth. This indicates that every newborn with potential HDN was detected at birth, and no one was readmitted after discharge due to HDN. The conclusion of a study conducted in 2023 suggests that paediatricians should ensure early treatment for hyperbilirubinemia in cases where sensitisation has been determined antenatally [14]. Thus, our twenty-five-year research period confirms this recommendation in practice, as only early determination of sensitisation and laboratory monitoring of bilirubin have led to prompt and successful treatment, especially of ABO HDN, because the diagnostic criteria of ABO HDN are the subject of considerable debate and clinical confusion, especially due to the weaker positive DCT [15]. A review published in 2023 shows significant discrepancies between studies on HDN, and that the postnatal burden of HDN, including the need for neonatal interventions such as ET, remains severe in newborns [1]. The results of our long-term study show that screening during pregnancy and early identification of HDN have reduced the need for ET, with only 10 ETs performed in the last 10 years. Further study into how RhD disappeared from the scene and other alloimmunisations appeared in pregnancy and their consequences on the newborn should be done, as well as about alloimmunisation and its consequences as a global neonatal problem. This would raise awareness and knowledge about HDN, for which there is no universal cure [16]. The shortcomings of our study regarding the lack of data on immunoprophylaxis RhD, and more detailed and more precise laboratory tests for HDN, especially for ABO in newborns, should be an incentive for further research.

We conclude that in the region covered by our 25-year study, thanks to the excellent collaboration of the gynaecologists, transfusionists, and neonatologists who are responsible for the treatment of HDN, there has been an increase in immunohaematological screening occurrence and frequency and an increase in the detection of non-RhD alloimmunisation in pregnancy in the last decade. Therefore, constant monitoring is needed in this area, especially in the search for diagnostic and therapeutic guidelines for ABO immunisation and its consequences for the newborn, since a universal cure for HDN still does not exist.

### Key Messages

1. The diagnosis, and treatment of alloimmunisation in pregnancy and its consequences for newborns.
2. The diagnostic criteria of ABO HDN are the subject of considerable debate and clinical confusion, especially due to the weaker positive DCT.
3. The immunoprophylaxis is successful, the screening of pregnant women is almost comprehensive, and HDN is diagnosed at an early age with a low percentage of ET as a treatment model.
